# Mapping of PARK2 and PACRG Overlapping Regulatory Region Reveals LD Structure and Functional Variants in Association with Leprosy in Unrelated Indian Population Groups

**DOI:** 10.1371/journal.pgen.1003578

**Published:** 2013-07-04

**Authors:** Rupali Chopra, Shafat Ali, Amit K. Srivastava, Shweta Aggarwal, Bhupender Kumar, Siddharth Manvati, Ponnusamy Kalaiarasan, Mamta Jena, Vijay K. Garg, Sambit N. Bhattacharya, Rameshwar N. K. Bamezai

**Affiliations:** 1 Shri Mata Vaishno Devi University, School of Biotechnology, Katra, Jammu & Kashmir, India; 2 National Centre of Applied Human Genetics, School of Life Sciences, Jawaharlal Nehru University, New Delhi, India; 3 Department of Dermatology and Sexually Transmitted Diseases, Maulana Azad Medical College, Lok Nayak Jai Prakash Hospital, New Delhi, India; 4 Department of Dermatology and Venereology, University College of Medical Sciences and GTB Hospital, Delhi, India; Dartmouth College, United States of America

## Abstract

Leprosy is a chronic infectious disease caused by *Mycobacterium Leprae*, where the host genetic background plays an important role toward the disease pathogenesis. Various studies have identified a number of human genes in association with leprosy or its clinical forms. However, non-replication of results has hinted at the heterogeneity among associations between different population groups, which could be due to differently evolved LD structures and differential frequencies of SNPs within the studied regions of the genome. A need for systematic and saturated mapping of the associated regions with the disease is warranted to unravel the observed heterogeneity in different populations. Mapping of the PARK2 and PACRG gene regulatory region with 96 SNPs, with a resolution of 1 SNP per 1 Kb for PARK2 gene regulatory region in a North Indian population, showed an involvement of 11 SNPs in determining the susceptibility towards leprosy. The association was replicated in a geographically distinct and unrelated population from Orissa in eastern India. *In vitro* reporter assays revealed that the two significantly associated SNPs, located 63.8 kb upstream of PARK2 gene and represented in a single BIN of 8 SNPs, influenced the gene expression. A comparison of BINs between Indian and Vietnamese populations revealed differences in the BIN structures, explaining the heterogeneity and also the reason for non-replication of the associated genomic region in different populations.

## Author Summary

Leprosy is a chronic granulomatous infection caused by the intracellular organism *Mycobacterium leprae*. The disease affects the skin and the peripheral nerves and can cause irreversible impairment of the nerve function with consequent chronic disabilities. The prevalence of leprosy has declined dramatically after the introduction of Multidrug therapy in the 1980s. However, the infection continues to survive as a major public health problem with more than 200,000 new cases reported globally every year, especially in China and India. The disease is governed by host genetic background, where several genes have been identified in association with leprosy or its clinical forms. The involvement of the PARK2 and PACRG genes with leprosy susceptibility in two distinct populations of the world, Vietnamese and Brazilian, and its non-replication in other populations suggests unravelling the reasons of heterogeneity between different population groups. The possibility of involvement of other variants and a differential LD structure for the PARK2 regulatory region in Indian populations as compared to Brazilian and Vietnamese provides an answer to the heterogeneity among associations observed previously in different population groups.

## Introduction

*Mycobacterium leprae* is the causative agent of chronic granulomatous infectious disease, known as Leprosy. The disease affects skin, the peripheral nerves and can cause irreversible impairment of the nerve function with consequent chronic disabilities [1]. The prevalence of leprosy which declined dramatically after the introduction of Multidrug therapy in 1980s, however, continues to survive as a major public health problem with more than 200,000 new cases reported globally every year, especially in China and India [2]. Our understanding about the mechanism underlying infection and how it leads to different clinical forms is limited; because *M. leprae* only infects humans and cannot be cultured *in vitro* [3]. Only a limited number show clinically recognizable lesions [4], and a simultaneous spectrum of the disease symptoms that depends upon the interaction between host immune system and the pathogen. Tuberculoid and lepromatous leprosy are at opposite ends of the spectrum, associated with an immune response mediated either by type 1 helper T (Th1) or type 2 helper T (Th2) cells [5]. The limited genetic diversity between different isolates of *M. leprae* strains [6] illustrates that the differences in susceptibility towards the disease or its clinical manifestations among patients are governed by host genetic factors, which have been implicated from studies of familial clustering [7], studies of twins [8], complex segregation analysis [9], [10], and test of analysis with the HLA genes [11]. Recent genome-wide association studies [12], [13] have further supported the involvement of host genetic background in inter-individual variability. Several studies have identified a number of human genes, such as HLA-DR [14], [15], LTA [16], TLRs [17], [18]; and genomic regions like 10p13 [19], 6p21 [20], 17q11–q21 [21], 20p13 [22] and 6q25-26 harbouring variants in the common regulatory region of PARK2 and PACRG genes [23] to be associated with the disease or its clinical forms. The results have suggested a polygenic nature of the disease with a high degree of heterogeneity among different populations and only a few unequivocal replications.

PARK2 and PACRG genes both share a common regulatory region and encode the proteins that are involved in cellular ubiquitination. Little is known about the specific function of the PACRG gene. PARK2 protein product-parkin, however, has been identified as an ubiquitin E3 ligase involved in delivery of polyubiquinated proteins to the proteasomal complex [24]. Only experimental evidence for the involvement of the PARK2 and its co-regulated gene PACRG with the host responses to *M. leprae* was provided by positional cloning in Vietnamese and Brazilian populations [23]. Different pathway analyses also showed the importance of these genes in pathogenesis of the disease [13], [25]. However, attempts to replicate the results in other populations failed in the past [26], [27], suggesting the possible involvement of different variants in diverse populations providing susceptibility towards leprosy. This possibility could arise due to a change in LD structures across the populations for the SNPs distributed in the specific genomic regions.

The present study with this rationale selected a group of SNPs, saturating the regulatory region of PARK2 and PACRG genes, to find out the variant LD structure, if any, in Indian population as compared to Brazilian and Vietnamese; and study the unexplored variants that may be responsible for an association with leprosy or its sub-types in the studied population.

## Results

PARK2 and PACRG gene regulatory region was saturated with 96 SNPs with approximately 1 SNP per Kb for PARK2 gene regulatory region to perform a population based case-control study in two unrelated Indian population groups. To rule out population stratification in the studied groups which confounds a disease association study, the MDS (multi-dimensional scaling) plot based on IBS (identity by state) pair-wise distances for 61 individual identifying autosomal SNPs not associated with the disease [28] was carried out. The results showed a compact cluster indicating the populations under study to be homogeneous (Figure S1). Locus wise F_ST_ was also calculated for the SNPs associated with Leprosy in the Indian populations. All the polymorphisms showed a very low locus-wise F_ST_ value, indicating that the patients and controls belonged to the same population group.

**Figure 1:**
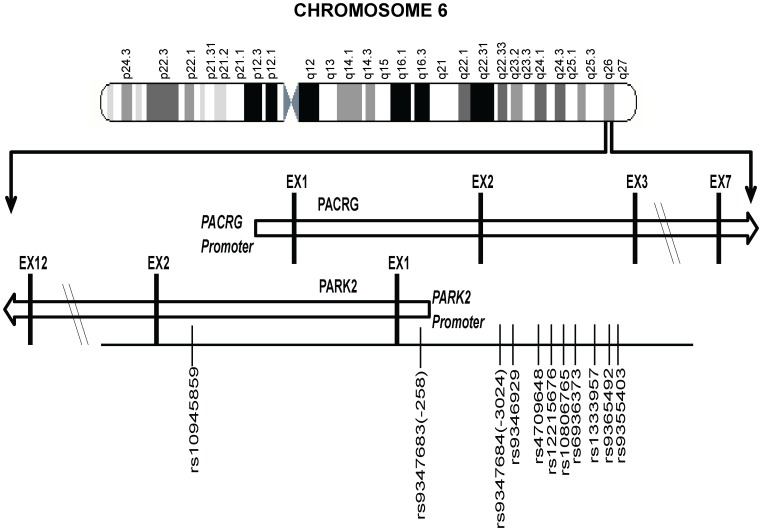
A partial map of Chromosome-6q26 expanded to show the position and distribution of 11 significant SNPs (shown with rs numbers) in the regulatory region of the PARK2 and PACRG genes.

Figure 1 provides a schematic picture of the distribution of 11 significantly associated SNPs out of a total of 96 SNPs studied for the region (criteria details provided in the Materials and Methods section) in two geographically distinct and unrelated population groups, using a MassArray platform. Detailed distribution, minor allele frequencies, HWE status and BIN structure information for all studied SNPs in controls and patients is provided in Table S1; and the information on 11 significantly associated SNPs along with their ORs and *P* values are presented in Table 1.

**Table 1:** Allele and genotype frequencies for 11 significant SNPs within PARK2 and PACRG gene regulatory region in two different cohorts of patients with Leprosy.

		North India			Orissa Population			Combined					Logistic p Value			
SNPs	Risk Allele	Control Frequency	Patient Frequency	p^a^ value	Control Frequency	Patient Frequency	p^b^ value	Control Frequency	Patient Frequency	PB Frequency	MB Frequency	p^c^ value	Model	Unadjusted p^d^ value	Adjusted p^e^ value	OR(95%CI)
rs10945859	Minor-C	0.28	0.33	0.00020	0.16	0.23	0.013	0.26	0.31	0.31	0.32	0.00012, 0.0075, 0.00058	CT+CCvsTT	0.00053, 0.015, 0.0022	0.0039, 0.06, 0.0078	1.32(1.12–1.54), 1.29(1.05–1.59), 1.35(1.11–1.64)
rs9347683	Minor-C	0.28	0.33	0.00024	0.16	0.23	0.017	0.26	0.31	0.31	0.32	0.00018, 0.0094, 0.00075	CA+CCvsAA	0.00077, 0.015, 0.0035	0.0056, 0.062, 0.013	1.31(1.12–1.53), 1.29(1.05–1.59), 1.33(1.09–1.62)
rs9347684	Minor-C	0.27	0.31	0.0015	0.14	0.22	0.0057	0.25	0.30	0.30	0.30	0.00081, 0.013, 0.0043	CT+CCvsTT	0.0014, 0.01, 0.011	0.0083, 0.041, 0.034	1.29(1.1–1.51), 1.31(1.06–1.62), 1.28(1.05–1.56)
rs9346929	Minor-A	0.28	0.33	0.00066	0.15	0.23	0.0047	0.27	0.31	0.31	0.32	0.00033, 0.014, 0.0012	AG+AAvsGG	0.00064, 0.015, 0.0029	0.0047, 0.058, 0.01	1.31(1.12–1.54), 1.29(1.05–1.6), 1.34(1.1–1.63)
rs4709648	Minor-C	0.37	0.42	0.0014	0.22	0.31	0.0064	0.35	0.40	0.40	0.39	0.00067, 0.0042, 0.0092	CG+CCvsGG	0.012, 0.037, 0.051	0.055, 0.12, 0.13	1.23(1.04–1.44), 1.25(1.01–1.56), 1.21(0.99–1.48)
rs12215676	Minor-G	0.36	0.42	0.00021	0.22	0.31	0.0079	0.34	0.40	0.40	0.39	0.00012, 0.0015, 0.0026	CG+GGvsCC	0.0022, 0.01, 0.021	0.013, 0.032, 0.067	1.28(1.09–1.51), 1.33(1.07–1.65), 1.26(1.03–1.54)
rs10806765	Minor-T	0.28	0.33	0.00068	0.15	0.23	0.0039	0.27	0.31	0.31	0.32	0.00031, 0.01, 0.0015	TC+TTvsCC	0.00053, 0.012, 0.0029	0.0039, 0.045, 0.01	1.32(1.12–1.55), 1.31(1.06–1.61), 1.34(1.1–1.63)
rs6936373	Minor-G	0.36	0.42	0.00040	0.22	0.31	0.0089	0.35	0.40	0.40	0.39	0.00022, 0.0019, 0.0045	GC+GGvsCC	0.0043, 0.021, 0.023	0.023, 0.067, 0.07	1.26(1.07–1.48), 1.28(1.03–1.59), 1.26(1.03–1.53)
rs1333957	Minor-A	0.28	0.33	0.00031	0.15	0.23	0.0031	0.26	0.31	0.31	0.31	0.00013, 0.0064, 0.00078	AC+AAvsCC	0.00042, 0.0092, 0.0027	0.0034, 0.042, 0.0095	1.32(1.13–1.55), 1.32(1.07–1.62), 1.34(1.1–1.63)
rs9365492	Minor-C	0.27	0.33	0.000040	0.15	0.23	0.0038	0.25	0.31	0.30	0.32	0.000015, 0.0033, 0.000081	CT+CCvsTT	0.000034, 0.0033, 0.0003	0.00036, 0.013, 0.0015	1.39(1.19–1.63), 1.36(1.11–1.68), 1.43(1.17–1.74)
rs9355403	Minor-A	0.28	0.32	0.00051	0.15	0.23	0.0026	0.26	0.31	0.30	0.31	0.00018, 0.013, 0.00056	AG+AAvsGG	0.00062, 0.021, 0.0019	0.0047, 0.077, 0.0072	1.31(1.12–1.54), 1.27(1.03–1.57), 1.36(1.12–1.65)

**p^a^** and **p^b^** value for 2×2 chi test for overall allelic frequencies comparison of samples from North Indian and samples from Orissa. **p^c^** value for 2×2 chi test for overall allelic frequencies comparison of combined samples, PB and MB samples. **p^d^** and **p^e^** values for genotypic model by logistic regression for combines samples, PB and MB samples before and after adjustment for sex as a covariate. Bonferroni correction of 32 SNPs was applied for multiples testing. Out of total 96 SNPs tested 64 were in 14 bin set (r^2^>0.8) [data not shown].

Eleven of the studied 96 SNPs showed a consistent and strong association with leprosy susceptibility, both in the North Indian and the East Indian-Orissa population groups. Ten out of 11 SNPs were located in the regulatory region of the PARK2 gene and a single SNP within the regulatory region of the PACRG gene (Figure 1). The observation made for the 11 SNPs on 2305 samples (829 leprosy patients and 1476 controls) from northern India was also made in a geographically unrelated Indian population of 380 individuals (184 leprosy patients and 196 controls) from Orissa in East India with a consistent association for SNP rs10945859, located 6.67 kb upstream of PACRG gene, rs9347683 (−258) within the core promoter region of PARK2 gene and SNPs rs9347684 (−3024), rs9346929, rs4709648, rs12215676, rs10806765, rs6936373, rs1333957, rs9365492, rs9355403, located within 63.8 kb upstream region of the PARK2 gene.

A combined analysis of the North Indian and East Indian-Orissa population groups confirmed the strong association for these 11 SNPs: rs10945859 (CC+CT vs. TT, OR = 1.32, 95% CI = 1.12–1.54, *p* = 5.30E-04); rs9347683(−258) (CC+CA vs. AA, OR = 1.31, 95% CI = 1.12–1.53), *p* = 7.70E-04); rs9347684 (CC+CT vs. TT, OR = 1.29, 95% CI = 1.10–1.51, *p* = 1.40E-03), rs9346929 (AA+GA vs. GG, OR = 1.31, 95% CI = 1.12–1.54, *p* = 6.40E-04), rs4709648 (CC+CG vs. GG, OR = 1.23, 95% CI = 1.04–1.44, *p* = 1.20E-02), rs12215676 (GG+CG vs. CC, OR = 1.28, 95% CI = 1.09–1.51, *p* = 2.20E-03), rs10806765 (TT+TC vs. CC, OR = 1.32, 95% CI = 1.12–1.55, *p* = 5.30E-04), rs6936373 (GG+GC vs. CC, OR = 1.26, 95% CI = 1.07–1.48, *p* = 4.30E-043), rs1333957 (AA+CA vs. CC, OR = 1.32, 95% CI = 1.13–1.55, *p* = 4.20E-04), rs9365492 (CC+TC vs. TT, OR = 1.39, 95% CI = 1.19–1.63, *p* = 3.40E-05), rs9355403 (AA+GA vs. GG, OR = 1.31, 95% CI = 1.12–1.54, *p* = 6.20E-04). The association of all 11 SNPs, involving the minor allele for the risk, was strong even after adjustment with sex as a covariate and the Bonferroni correction for multiple testing. A stepwise multivariate logistic regression analysis for eleven significantly associated SNPs along with the sex as a covariate in combined population showed retention of 2 out of 11 SNPs (rs9365492, p = 0.0033 and rs9355403, p = 0.024) in the model. In addition, analysis after dividing the patients in two known sub-types of the disease, i.e., pauci-bacillary (PB) and multi-bacillary (MB), both within North Indian and East Indian-Orissa population, showed a strong association of all the 11 SNPs with PB and MB form of the leprosy with a power >98%, MAF = 0.27 and OR = 1.44 in the North Indian and >50%, MAF = 0.15 and OR = 1.55 for East Indian-Orissa population (Table S2). The association with the MB sub-type in comparison to the PB form of the disease showed higher significance values. However, the heterogeneity testing between the PB and MB form of the leprosy did not show any significant difference between the two groups.

### LD and Bin structure of studied SNPs in Indian population

**Figure 2:**
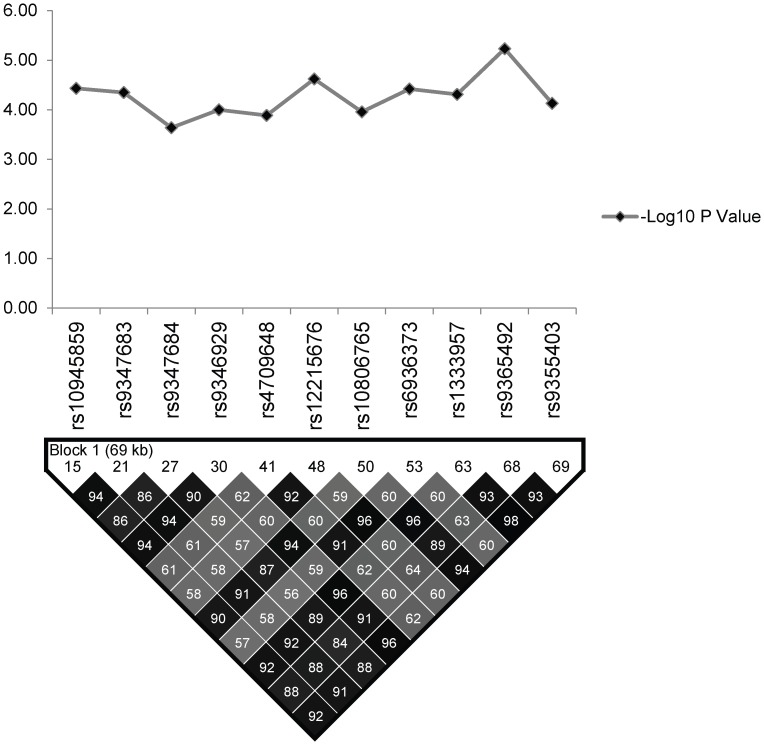
The association statistics of the 11 significant SNPs in the regulatory region of the PARK2 and PACRG genes; presented as negative logarithm of the *P*-Value and their linkage disequilibrium (LD) plot based on pairwise LD for r^2^ cut off value ≥0.80.

**Figure 3:**
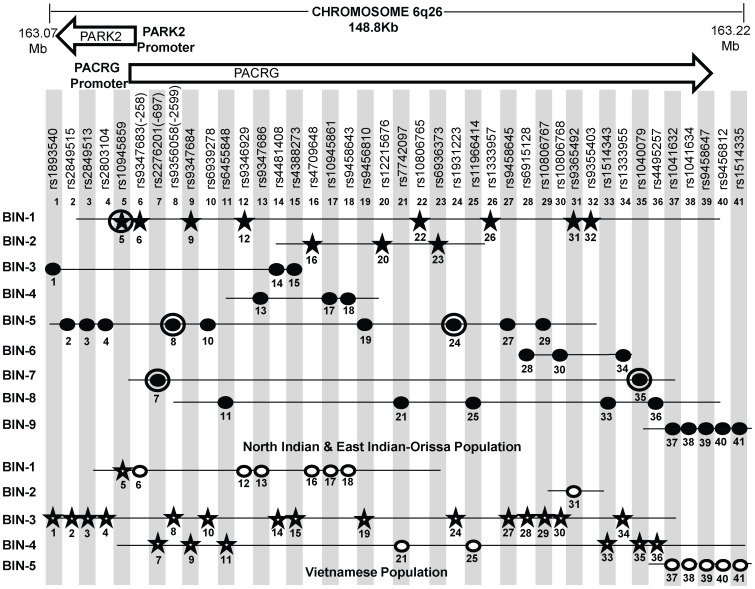
A schematic lay-out of the BIN structure (r^2^≥0.80) in the regulatory region of the PARK2 and PACRG genes in North Indian and East Indian-Orissa and Vietnamese population for 41 SNPs spanning 148 Kb region of Chromosome 6q26, where 36 SNPs are common to both Vietnamese and Indian population and 5 significant SNPs (No. 20, 22, 23, 26, 32) are exclusively studied in the Indian population. [It may be noted that a similar BIN structure was observed in the North-Indian and East-Indian-Orissa populations]. Physical location of the studied chromosomal region is given in Mb on top. Vietnamese population information of Mira et al, 2004 and common SNPs between Indian and Vietnamese population (Alter et al, 2012) was shared by Prof. Schurr. Rest of the SNPs & BIN structure information was retrieved from Alter et al (2012). SNPs in star shape indicate the significant association (in two respective populations-Indian and Vietnamese). 11 significantly associated SNPs in studied Indian populations are distributed in two BINs (BIN 1 with 8 and BIN 2 with 3 SNPs). Distribution of significant SNPs in Vietnamese population is shown in BIN 1, BIN 2 and BIN3. SNPs, rs10945859 (No. 1) and rs9347684 (No. 9), although shared significance in both the Indian and Vietnamese population, but these showed no significant difference in expression in *in vitro* reporter assay for the alternative alleles. Each SNP is designated by a No. ranging from 1 to 41 according to increasing order of the chromosomal position. Filled Black Star - Significant SNPs in Indian (North and East Indian-Orissa) population, Unfilled Star - Significant SNPs in Vietnamese population, Filled Black Dot - Non-Significant SNPs in North and East Indian-Orissa population, Unfilled Dot - Non-Significant SNPs in Vietnamese population, Black Circled Dot and Black Circled Star SNPs (No. 5, 7, 8, 24, 35) studied by us earlier [26].

Linkage Disequilibrium (LD) analysis of the studied SNPs in regulatory region of PARK2 and PACRG was performed using Haploview v4.2 in controls of North Indian and East Indian-Orissa population and compared with the Vietnamese. The detailed distribution of 96 SNPs in different BINs (for r^2^ cut off value ≥0.80) within North Indian and East Indian-Orissa population is provided in Table S1, which also includes 11 significantly associated SNPs as part of two BINs (BIN-1 with 8 and BIN-2 with 3 significantly associated SNPs) (Figure 2). The 3 significant SNPs (rs1333955, rs10806768, rs6915128) within our (North & East Indian–Orissa) and recently published North Indian (Agra) [29] study, grouped together in a single BIN-6 (Figure 3; Table S1), however, the significance in both the studies was marginal; and in our case was lost after Bonferroni correction.

In order to draw a parity between the studied SNPs for the overlapping regulatory region between PARK2 and PACRG genes in the Vietnamese and both groups of Indian populations (North and East Indian-Orissa), detailed information was sought for the Vietnamese samples. Information of 81 SNPs studied in the Vietnamese population [23] and 41 SNPs common between Indian (North, East Indian-Orissa) and Vietnamese as studied by Alter et al [29], was made available (courtesy Dr. Schurr) and rest of the studied SNP Bin structure information was retrieved from the supplementary files provided in the article. A comparison with 96 SNPs studied in Indians showed 36 SNPs common to both Vietnamese and Indian population and 5 significant SNPs exclusive to Indian population and not studied in the Vietnamese. The 5 SNPs were part of the 11 significantly associated SNPs observed in Indian samples; and the remaining 6 SNPs were part of the group of 36 SNPs common between Vietnamese and Indians. This allowed us to generate the BIN structure for 41 SNPs, which included 41 SNPs in Indian population and 36 SNPs for Vietnamese. The 11 significant SNPs observed in our (North & East Indian-Orissa) study were distributed in two BINs (8 in one BIN and 3 in another BIN) and rest of the 30 non-significant SNPs were distributed in seven other BINs. The BIN structure generated with the available information (Table S1) did not differ between our studied (North & East Indian-Orissa) samples and that of the North Indian (Agra) samples studied by the Alter et al [29] (data not shown).

However, the BIN structure generated for 36 SNPs in Vietnamese were distributed in five BINs (Figure 3). BIN-3 and BIN-4 in Vietnamese contained 15 and 8 SNPs, respectively to add up to 23, where 21 out of 23 SNPs were significantly associated in this population. However, 20 of these 21 SNPs were observed to be non-significant in Indian population groups studied, and constituted different BIN structures (BIN-3 to BIN-9). BIN-1 in Vietnamese population contained 7 SNPs, including the SNP rs10945859 located 6.67 kb upstream of PACRG gene, that was significantly associated both in Vietnamese and Indian population and BIN-2 of the Vietnamese population contained only single SNP, rs9365492. The 3 out of the 6 SNPs within the promoter region of PARK2 gene, located in BIN-1 and the single SNP, rs9365492 in BIN-2 were non-significant in Vietnamese population and showed significance in Indian population. Thus, comparing BIN-1 and BIN-2 in Vietnamese population with BIN-1 in Indian population, carrying 8 significantly associated SNPs; we found that 1 SNP in the BIN in Vietnamese and all the 8 SNPs in Indians showed a significant association with leprosy. However, the functional significance of the 2 common significant SNPs (rs10945859, rs9347684) between the two populations (Vietnamese and Indian) did not show any significant difference in expression in *in vitro* reporter assay for the alternative alleles (data not shown).

### Haplotype analysis

Haplotype analysis (Tables 2–4), using haplostats software-the Haplotype 4, with risk alleles at all the 11 significantly associated SNP positions, showed an increased risk (OR = 1.36, *p* = 2.46E-06, Freq_controls_ = 23%, Freq_patients_ = 29%) when compared to other haplotypes, generated for the 11 significantly associated SNPs in the combined Indian population (Table 2). A stepwise multivariate logistic regression analysis for 11 significantly associated SNPs (distributed in 2 BINs), keeping the sex as a covariate in combined Indian population, showed that 2 out of 8 SNPs (rs9365492, rs9355403) of BIN-1 were significant in the model. Thus BIN-1 remained most strongly associated with susceptibility to leprosy. Subsequently, we performed the phased analysis of SNPs in BIN-1 and BIN-2 to identify the haplotypes showing stronger association with leprosy (Tables 2–4). This was done to assay for combination of SNPs in either of the BINs providing more risk towards leprosy susceptibility. Haplotype 3 with risk alleles at all the 8 significantly associated positions provided an increased risk (OR = 1.34, *p* = 2.88E-06, Freq_controls_ = 23%, Freq_patients_ = 29%) in comparison to other haplotypes generated in the combined Indian population (Table 3). Similarly, BIN-2 representing the Haplotypes of 3 significantly associated SNPs showed Haplotype 2 with risk alleles at all the 3 significantly associated positions, providing an increased risk (OR = 1.29, *p* = 7.56E-06, Freq_controls_ = 34%, Freq_patients_ = 40%) in comparison to other haplotypes generated for the 3 significantly associated SNPs in the combined Indian population (Table 4).

**Table 2:** Haplotype structure, haplotype frequencies, significant *p* values and odds ratio between patients versus healthy controls of 11 significantly associated SNPs.

Haplotype	rs10945859	rs9347683	rs9347684	rs9346929	rs4709648	rs12215676	rs10806765	rs6936373	rs1333957	rs9365492	rs9355403	Hap-Score	p^a^-val	pool.hf	control.hf	case.hf	glm.eff	OR.lower	OR	OR.upper
1	T	A	T	G	G	C	C	C	C	T	G	−3.14	**1.69E-03**	0.62	0.63	0.59	Base	NA	1	NA
2	C	C	T	A	C	G	T	G	A	C	A	0.20	8.40E-01	0.01	0.01	0.01	Eff	0.70	1.16	1.93
3	T	A	T	G	C	G	C	G	C	T	G	0.41	6.84E-01	0.08	0.08	0.09	Eff	0.90	1.12	1.40
4	C	C	C	A	C	G	T	G	A	C	A	4.71	**2.46E-06**	0.25	0.23	0.29	Eff	1.19	1.36	1.56
5	C	A	C	A	C	C	T	C	A	C	A	NA	NA	0.00	0.00	NA	<NA>	NA	NA	NA

Column: Hap-Score shows haplotype score statistic; Base, part of the baseline; Frequencies and disease association of haplotype of SNP alleles was tested using *haplo.cc* extended application of Haplo.stasts software (v1.4.4) which combines the results of *haplo.score*, *haplo.group* and *haplo.glm*. Haplotype frequency was computed by maximum likelihood estimates of haplotype probabilities with progressive insertion algorithm and *haplo.cc* computed score statistic to test association between haplotype and traits with adjustment for non-genetic covariates (sex).

p^a^ Indicates the haplotype comparison statistics for patients vs controls.

**Table 3:** Haplotype structure, haplotype frequencies, significant *p* values and odds ratio between patients versus healthy controls of 8 SNPs representing BIN-1 of Indian population.

Haplotype	rs10945859	rs9347683	rs9347684	rs9346929	rs10806765	rs1333957	rs9365492	rs9355403	Hap-Score	p^a^-val	pool.hf	control.hf	case.hf	glm.eff	OR.lower	OR	OR.upper
1	T	A	T	G	C	C	T	G	−3.33	**8.55E-04**	0.71	0.72	0.68	Base	NA	1.00	NA
2	C	C	T	A	T	A	C	A	0.24	8.07E-01	0.01	0.01	0.01	Eff	0.70	1.17	1.94
3	C	C	C	A	T	A	C	A	4.68	**2.88E-06**	0.25	0.23	0.29	Eff	1.18	1.34	1.53
4	C	A	C	A	T	A	C	A	NA	NA	0.00	0.00	NA	<NA>	NA	NA	NA

Column: Hap-Score shows haplotype score statistic; Base, part of the baseline; Frequencies and disease association of haplotype of SNP alleles was tested using *haplo.cc* extended application of Haplo.stasts software (v1.4.4) which combines the results of *haplo.score*, *haplo.group* and *haplo.glm*. Haplotype frequency was computed by maximum likelihood estimates of haplotype probabilities with progressive insertion algorithm and *haplo.cc* computed score statistic to test association between haplotype and traits with adjustment for non-genetic covariates (sex).

p^a^ Indicates the haplotype comparison statistics for patients vs controls.

**Table 4:** Haplotype structure, haplotype frequencies, significant *p* values and odds ratio between patients versus healthy controls of 3 SNPs representing BIN-2 of Indian population.

Haplotype	rs4709648	rs12215676	rs6936373	Hap-Score	p^a^-val	pool.hf	control.hf	case.hf	glm.eff	OR.lower	OR	OR.upper
1	G	C	C	−3.56	**3.74E-04**	0.63	0.65	0.59	Base	NA	1.00	NA
2	C	G	G	4.48	**7.56E-06**	0.36	0.34	0.40	Eff	1.14	1.29	1.45
3	C	C	C	NA	NA	0.01	0.01	0.00	R	0.20	0.39	0.76

Column: Hap-Score shows haplotype score statistic; Base, part of the baseline; Frequencies and disease association of haplotype of SNP alleles was tested using *haplo.cc* extended application of Haplo.stasts software (v1.4.4) which combines the results of *haplo.score*, *haplo.group* and *haplo.glm*. Haplotype frequency was computed by maximum likelihood estimates of haplotype probabilities with progressive insertion algorithm and *haplo.cc* computed score statistic to test association between haplotype and traits with adjustment for non-genetic covariates (sex).

p^a^ Indicates the haplotype comparison statistics for patients vs controls.

### Luciferase expression study for the SNPs significantly associated with the disease

Out of 11 significantly associated SNPs with leprosy in Indian population, only one core promoter SNP rs9347683 (−258) of PARK2 gene had been analysed functionally and documented in literature [30], [31]. None of the other SNPs in the region were studied earlier for their functional implication. The 2 SNPs (rs9365492 and rs9355403), 113 bp apart, lying within 63.8 kb upstream region of PARK2 gene; and two SNPs found significant in both Indian and Vietnamese population, SNP rs9347684 located within the 3.5 kb upstream region of the PARK2 gene and another SNP rs10945859 located 6.67 kb upstream of PACRG gene were chosen to assay their functional role and were cloned in the pGL3 promoter bearing luciferase-reporter expressing vector.

**Figure 4:**
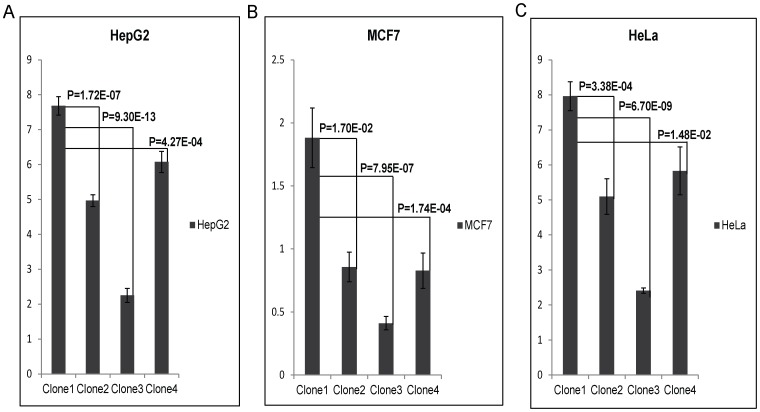
Luciferase expression assay of upstream SNPs of PARK2 gene (rs9365492 (T/C) and rs9355403 (G/A)): C & A respectively represent risk allele for the SNP. Bar with standard error shows the mean expression values in three different cell lines (HepG2, MCF7 and HeLa) for different Clones in PGL3 promoter vector: Clone1, with protective allele combination - rs9365492(T)-rs9355403(G); Clone2, with risk and protective allele combination - rs9365492(C)-rs9355403(G); Clone3, with protective and risk allele combination - rs9365492(T)-rs9355403(A) and Clone4, with risk allele combination - rs9365492(C)-rs9355403(A). *P*-Values for comparison of mean (one way ANOVA) expression between clones with different allele combination of 2 SNPs is also shown.

To test the enhancer activity of the SNPs, rs9365492 and rs9355403, the region bearing both the SNPs were cloned in pGL3 promoter vector in 4 allele combinations (Table S3). All 4 clones were transfected in 3 different cell lines: HepG2, MCF7 and HeLa. The result showed a lower expression for Clone2, Clone3 and Clone4 compared to Clone1 containing both SNPs as protective alleles (Figure 4). The expression was lowest in Clone 3 with rs9365492(T)-rs9355403(A), representing protective allele for SNP rs9365492 and risk allele for rs9355403. Bioinformatics analysis, using Tansfac-AliBaba2 tool [32] and HaploReg [33] (Collection from TRANSFEC, JASPER and protein-binding microarray experiments) databases revealed that the minor Risk alleles for both the SNPs, rs9365492 and rs9355403, affected the transcription binding site (Table S3).

SNP-rs9347684, located 3.5 kb upstream region of the PARK2 gene; and SNP-rs10945859, located within the 6.67 kb upstream region of PACRG gene, were cloned in pGL3 promoter vector to test for enhancer activity. Clone1 with rs9347684 protective T allele, Clone2 with risk C allele and similarly Clone1 with rs10945859 protective T allele and Clone2 risk C allele, did not show any significant change in the reporter gene expression in any of the 3 cell lines (data not shown).

## Discussion

Leprosy continues to remain a major health problem in many parts of the world, regardless of long history of research, advances in the medical field and the introduction of Multidrug therapy (MDT) in 1980s. The inability to grow the bacterium *in vitro* has been one of the inadequacies to unravel the intricacies of the biology of the disease. Yet efforts have been made to identify the role of host genetic factors to understand susceptibility mechanisms, especially in the background of limited genetic diversity between different isolates of *M. leprae*. Research has progressed over the years in identifying many candidates as risk providers, using genome wide linkage, association and candidate gene studies. However, search for common genetic variants across the afflicted population groups in the world has emerged equivocal. Looking for genes and its variants which are proposed either by genome wide linkage or association studies with an assumed importance in the pathway biology of the disease does provide a window for re-search. More so when the LD maps for the relevant genomic regions are expected to differ from one population group to another, explaining the heterogeneity among associations.

The present study fine mapped the overlapping PARK2 and PACRG gene regulatory region to detect the variant(s) associated with Leprosy susceptibility in geographically distinct and unrelated Indian population groups. Since earlier studies did not succeed in replicating [26], [27] the association of the studied variants within this shared region of the genes with Leprosy; as was observed in Vietnamese and Brazilian population [23], it was pertinent to re-visit the region with sufficiently saturated number of SNPs. The purpose was to unravel any difference in LD structures and the heterogeneity in association in-between population groups. This assumption was based on the fact that involvement of PARK2/PACRG which made some relevance in understanding the patho-biology of leprosy in two unrelated populations of the world, i.e. Brazilians and Vietnamese, should have shown its involvement in the disease even in different ethnic groups of India, despite heterogeneity in association. If this were true, the nature of heterogeneity could be explained through differential LD structures, involving variants within the same gene. To answer this question it was appropriate to study the overlapping regulatory region saturated with 96 SNPs (nearly 1 SNP/Kb for PARK2 regulatory region) and compare the LD structure between the Indian and Vietnamese population.

The LD map of 96 SNPs (Table S1) in two geographically distinct and unrelated populations of India, included 2 BINs of the 11 Significant SNPs (Figure 2). Further, a comparison of Haplotypes generated with 11 significant SNPs associated with leprosy in Indians showed that Haplotype 4 (Table 2) with risk alleles at all the 11 SNP loci provided an increased risk (OR = 1.36, *p* = 2.46E-06) when compared to the Haplotypes generated (Table 3 and Table 4) after categorizing the 11 significant SNPs on the basis of BINs; BIN-1 with 8 and BIN-2 with 3 SNPs. The haplotype analysis and the expression profile for the studied significant SNPs in the PARK2 gene regulatory region confirmed that the risk allele for the significantly associated SNPs were responsible for an increased risk towards leprosy and the same risk SNP allele disrupted the transcription factor binding site in a bioinformatics analysis, confirmed further by a reduction in expression in an *in-vitro* reporter (luciferase) expression analysis.

In order to compare the SNP distribution within the overlapping regulatory region in Vietnamese and Indians, an LD map was generated of 41 SNPs, with 36 common to Indians and Vietnamese and 5 exclusive to Indians and not studied in Vietnamese (Figure 3, Table S1). Confining to the number of these SNPs, instead of what actually could have been compared, was due to the availability of the information in Vietnamese (courtesy Dr. Schurr) [23], [29]. Most of the significant SNPs in Vietnamese population were located in the region far below 3′ side of the PARK2 gene [29] and not located in the regulatory region of the PARK2 and the PACRG, the focus of our study. However, Alter et al [29] in their study found 3 SNPs (rs1333955, 10806768, rs6915128) located in the regulatory region of the PARK2 to be significantly associated both in Vietnamese and Indian (Agra) population. The same SNPs were found significant by us as well but the significance was marginal (Table S1) and was lost after Bonferroni correction. Also, the 2 SNPs rs10945859 and rs9365492 studied by Alter et al [29], representing Indian population of Agra, were common to our 11 significant SNPs in north and east India-Orissa populations, however, these did not turn out to be significant in Agra population studied from India. The reason possibly is the small sample size of their studied Indian (Agra) population or presumably some unknown methodological reason. We have confronted a similar experience earlier where we could not replicate the significant association of rs10945859 (Malhotra et. al. [26]) in leprosy susceptibility; and do find its involvement in a larger sample set using MassArray genotyping procedure. Further, having an information on missing SNPs in Vietnamese would provide in future an exact BIN structure for the regulatory region for comparison with information available from other and diverse Indian populations; which would throw additional light on the evolution of LD structures and the differences in unrelated populations, such as Vietnamese, Brazilian, Chinese, Indians, where heterogeneity among association for the genes have been reported for Leprosy disease. Incidentally, as expected all the studied samples from India either by us (North Indian comprising Delhi, U.P., Bihar and East Indian-Orissa) or Alter et al (Agra) showed an overlapping BIN structure with the available SNP information (Table S1) which differed from that of Vietnamese [23], [29] (Figure 3). Thus, there are no discrepancy in-between population groups within Northern part of India at least. The observations also replicated in East Indian-Orissa population with a power >50% of association, which could further increase with the increase in sample size. The homogeneity check using 61 individual identifying autosomal SNP markers [28] for the studied North Indian and East Indian-Orissa populations showed a compact cluster, suggesting the homogeneity between the studied populations (Figure S1). Moreover, a similar BIN structure was observed in the North-Indian and East Indian-Orissa populations. However, interestingly the variation in LD structure between the Indian and the Vietnamese population was apparent as one of the causes of genetic heterogeneity.

A comparison of the 36 common SNPs between Indian and Vietnamese population for the region, generated different BIN structures in the two populations (Figure 3). The 20 significant SNPs in Vietnamese population could not be replicated in Indians (Figure 3), supporting the heterogeneity in association in the two unrelated populations of the world. Also, the analysis of 2 common significant SNPs in-between Indian and Vietnamese populations, rs9347684 (3.5 kb upstream of the PARK2 gene) and rs10945859 (6.67 kb upstream of the PACRG gene), both part of 8 significant SNPs in BIN-1 in Indians, failed to show any functional significance in *in-vitro* reporter (luciferase) expression profiles obtained for the alternative variants. This probably suggests that the two potential SNPs common to the two populations do not have any functional bearing on the biological process critical to the disease development. The remaining 4 SNPs (rs9347683, rs9346929, rs4709648, rs9365492) out of 36 common SNPs with a significant association only in Indian population were part of BIN-1 and BIN-2 containing 11 significantly associated SNPs. Among these, 1 SNP of BIN-1 has been functionally defined as a core promoter SNP rs9347683 (−258) [30], [31]. The functional importance of this SNP was also reflected in the HaploReg database [33] (collection from TRANSFEC, JASPER and protein-binding microarray experiments). To find out if there was any other functional SNP within BIN-1 in Indian population to explain the heterogeneity among the populations, we selected most significant SNP, rs9365492 and another SNP 113 bp apart, rs9355403, one of these rs9365492 located in a separate BIN-2 in Vietnamese population and the other rs9355403 not studied by them [29], [30]. When checked through Bioinformatics analysis, the SNP positions were involved in the transcription factor binding (Table S3). Further comparison of both these SNPs with the close primates (Chimpanzee, Orangutan, Rhesus, Gorilla, Gibbon, Baboon), showed that the risk allele was absent in all the organisms and evaluation of allele frequencies between different population groups of the world showed the lowest frequency of the risk allele in the ancestral African population which kept increasing from European to Indians and Japanese (Table S4). *In vitro* reporter assays confirmed the involvement of the risk alleles in an enhancer like activity. The four possible haplotypes (Clones-1 to 4) of the two SNPs (rs9365492 and rs9355403) showed lower expression of the reporter gene for Clones 2 to 4 possessing risk alleles for either of the SNPs, when compared to Clone-1 (with protective alleles at both the SNP positions) (Figure 4). Lowest expression was observed for Clone-3. However, the expected combinations as designed in Clone-2 and Clone-3 of the haplotypes, were not observed in the patient and control samples studied. The overall analysis indicated a stronger repressing effect of the risk SNP allele rs9355403 in presence of the protective SNP allele rs9365492 in a haplotype when compared to other haplotype combinations. It is apparent from the differential expression results expected of PARK2 gene due to the SNP variations, how important it could turn out in driving immunological response against the bacterium in the primary host within Schwann cells and monocyte derived macrophages; by involving specific transcription factors in regulating the gene expression [23], which could further be validated in future studies by carrying out mobility/gel shift assays that would establish the exact role of theses SNPs in affecting transcription binding unequivocally.

Researchers have demonstrated *parkin* protein as a multi-functional protein with a likely role in proteolysis of damaged proteins. Other functions include its role in general protein turnover and several cellular functions as divergent as, cell cycle control, apoptosis and maintenance of mitochondrial function [34], [35]. Microarray expression of Drosophila *parkin* k/o model [36] has shown an increased expression of innate immune response genes. This indicates that *parkin* also plays an important role as an immune-regulatory molecule that contributes to down regulation of the immune responsiveness. In our study variant allele in the regulatory region of PARK2 gene is expected to reduce the expression of *parkin* protein, which in turn could contribute to the higher expression of the immune regulatory molecules [36]. The role of *parkin* protein in regulating the degradation of proteins involved in the immune response to *M. leprae* [37]–[39], support the preferential involvement in the susceptibility to multi-bacillary form of leprosy, as observed by us. Also various E3 ubiquitin ligase proteins act as suppressor molecules that limit IL-2 production and proliferation in anergic T-cell [40]. This conclusion is further supported by the fact that ubiqutin protein involved in the ubiqutination process is known to inhibit the production of the pro-inflammatory cytokine TNF-alpha and enhance the production of IL-4, IL-10, and IL-13 [41]–[46] leading to decreased CMI response towards the infectious agent. However, the mechanism underlying these effects need further work.

## Materials and Methods

### Ethics statement

The study was approved by the Institutional Ethics Review Board of JNU, as per the guidelines of Indian Council of Medical Research, India.

### Subjects

A study was carried out in 2685 samples from two different cohorts (including 829 Leprosy patients from North India; 184 Patients from Orissa, in Eastern India; 1476 unrelated healthy control subjects from northern India; and 196 unrelated healthy control subjects from Orissa, Eastern India). Northern Indian samples were collected from Lok Nayak Jai Prakash Hospital, New Delhi, and from Guru Teg Bahadur Hospital, Delhi, and the Orissa (Eastern Indian) samples were collected from Cuttack Leprosy Home and Hospital, Orissa. Diagnosis of Leprosy was made by at least 2 independent leprologists after a physical examination of each patient and standard histological and pathological examination of the affected skin lesions. The patients group was classified as pauci-bacillary (PB) or multi-bacillary (MB) according to the Ridley and Jopling criteria [47]. The present study includes 452 Pauci-bacillary patients and 560 Multi-bacillary patients, with a mean age of 32.30±3.2 years (range 6–80 years). All these patients were under treatment with multidrug therapy (MDT) specific for multibacillary (MB) and paucibacillary (PB) leprosy, as recommended by the World Health Organization.

The study included the Control group with mean age of 35.97 years (range 3–82 years). None of the controls had any family history of tuberculosis, leprosy or any other related disease. A pre-informed written consent form, following the Indian Council of Medical Research (ICMR) norms, was obtained from all individuals whose blood sample was collected.

### SNP selection and genotyping

To rule out the population stratification, we selected 61 individual identifying autosomal SNP markers [28] based on threshold heterogeneity >35%; Fst valve <0.06; Linkage Disequilibrium value (D')<0.011 and distribution among 52 different world populations.

To unravel the role of PARK2 and PACRG genes and to determine the contributory functional variants for leprosy susceptibility in the Indian population, we selected 96 SNPs from the shared regulatory genomic region of both the genes with a saturation of nearly 1 SNP per Kb for PARK2 gene regulatory region. SNP selection was carried out based on their minor allele frequency (>5%) in the publicly available database from the National Center of Biotechnology Information (NCBI) EntrezSNP (build 36) and the International HapMap project: [Han Chinese, Japanese (Asian populations), and African (Ancestral)] populations. SNPs were also included from the promoter, exonic, intronic boundary; and also chosen on the basis of their functional role as reported in literature.

The flanking sequences for all the SNPs were downloaded from the National Center of Biotechnology Information (NCBI) site. High-throughput genotyping of the SNPs was performed by the iPLEX Gold chemistry on the matrix-assisted laser desorption, ionization time of flight mass spectrometer (MALDI-TOF-Sequenom). SNPs with a call rate <90% were removed from the analysis. All the Significant SNPs had a call rate of >95%.

### Statistical analysis

SNP genotype frequencies were subjected to Hardy-Weinberg equilibrium (HWE) analysis in patients and controls. SNPs with deviation (p<0.01) from HWE were removed from the study. Significant association of SNPs was tested by 3×2 and 2×2 Chi-square test for overall genotype and allele frequencies between leprosy patients and controls. SNPs with overall significance (p<0.05) were also confirmed by unconditional logistic regression analysis for different genotype models (recessive, dominant and co-dominant) and then corrected for age and sex. Bonferroni correction was also applied for multiple testing. SPSS software, version 17 (SPSS) was used for statistical analysis.

Frequencies and disease association of haplotypes was tested using *haplo.cc* extended application of Haplo.stasts software (v1.4.4). Linkage disequilibrium (LD) structure was determined using Haploview software, (version 4.2) [48]. To ensure adequate quality in statistical results in an association study, power of the study was calculated by Quanto software (v1.2.4.0) for the combined samples from Delhi and Orissa based on allele frequency and the effective size of the respective polymorphism.

To lower the risk of population stratification, MDS (multi-dimensional scaling) analysis was carried out, using Plink software, version 1.06 [49], [50]. For population differentiation analysis, Fst was calculated by the formula {F_ST_ = (H_T_−H_S_)/H_T_}, where H_S_ and H_T_ are the global heterozygosity indices over subpopulations (patients, control subjects, and 4 HapMap populations) and total population.

### *In vitro* reporter expression analysis

Out of total 11 significantly associated SNPs, SNP rs10945859 located 6.67 kb upstream of the regulatory region of the PACRG gene, SNP rs9347684 located within the 3.5 kb upstream region of the PARK2 gene and two SNPs (113 bp apart) located within a 63.8 Kb upstream region of PARK2 gene, were assessed for their enhancer like activity. Amplicons of 633 bp bearing SNP rs10945859, 608 bp region containing SNP rs9347684 and 760 bp region containing the two SNPs (rs9365492 and rs9355403) were cloned into PGL3 promoter vector (Promega) carrying SV40 promoter and luciferase expression unit. Different combinations of SNP alleles were created into the PCR product and cloned into the vector to test for the functional analysis. SDM (site directed mutagenesis) was performed by the Stratagene mutagenesis kit. Phusion DNA polymerase (Finnzymes, Keilaranta, Espoo, Finland) was used for PCR amplification as well as for SDM of the cloned regions. Sequences of all the cloned inserts were confirmed by direct sequencing (Table S5). Plasmid DNA was isolated using the plasmid maxi kit (Qiagen Inc., Valencia, CA, USA) for transient transfection. ESCORT transfecting reagent was used to transfect HepG2, MCF7 and HeLa cells at a density of 1×10^5^ cells per well in twelve-well plates and grown in Dulbecco-modified Eagle medium with 10% bovine calf serum overnight, prior to transfection. A total of 1 µg of vector construct and 0.1 µg of pRL-TK *Renilla* luciferase vector (Promega Corporation) with 2 µL of Escort (Sigma) were used for each transfection. Cells were collected 48 h after transfection and analyzed using the Dual-Luciferase Reporter Assay System (Promega). Luciferase activity was detected by luminometer (TD-20/20, DLReady; Turner Designs, Inc., Sunnyvale, CA, USA, and Promega Corporation). The pRL-TK vector that provided the constitutive expression of Renilla luciferase was co-transfected as an internal control to correct the differences in both transfection and harvest efficiencies. Transfections were carried out in triplicates and repeated at least thrice in independent experiments. Mean luciferase activity for the alleles of SNP was compared by one way ANOVA and presented in a bar diagram along with standard error.

## Supporting Information

Figure S1 Three dimensional scatter plot showing homogeneity among North Indian and East Indian-Orissa samples. This plot is based on three components generated by principal component analysis.
(TIF)

Table S1 Details of 96 SNPs studied in PARK2 and PACRG gene regulatory region in Indian samples (North & East Indian-Orissa population).
(DOC)

Table S2 Allele frequencies for 11 significant SNPs within PARK2 and PACRG gene regulatory region along with their respective *P* Values and Odds Ratio in East Indian-Orissa population.
(DOC)

Table S3 Allele combinations generated in four Clones for 2 SNPs, located upstream of the PARK2 gene regulatory region, and their Bioinformatics prediction for transcription factor binding by using TRANSFEC and HaploReg databases.
(DOC)

Table S4 Allele frequency comparison of 2 Significant SNPs (rs9365492 and rs9355403) between different HapMap and our North-Indian populations.
(DOC)

Table S5 Primer sequences along with restriction sites for cloning specific regions of PARK2 and PACRG.
(DOC)
