# Supplementary figures and images for: Mapping of PARK2 and PACRG Overlapping Regulatory Region Reveals LD Structure and Functional Variants in Association with Leprosy in Unrelated Indian Population Groups
Source: PLoS Genet. 2013 Jul 4;9(7):e1003578. doi: 10.1371/journal.pgen.1003578 (PMC3701713; doi:10.1371/journal.pgen.1003578)

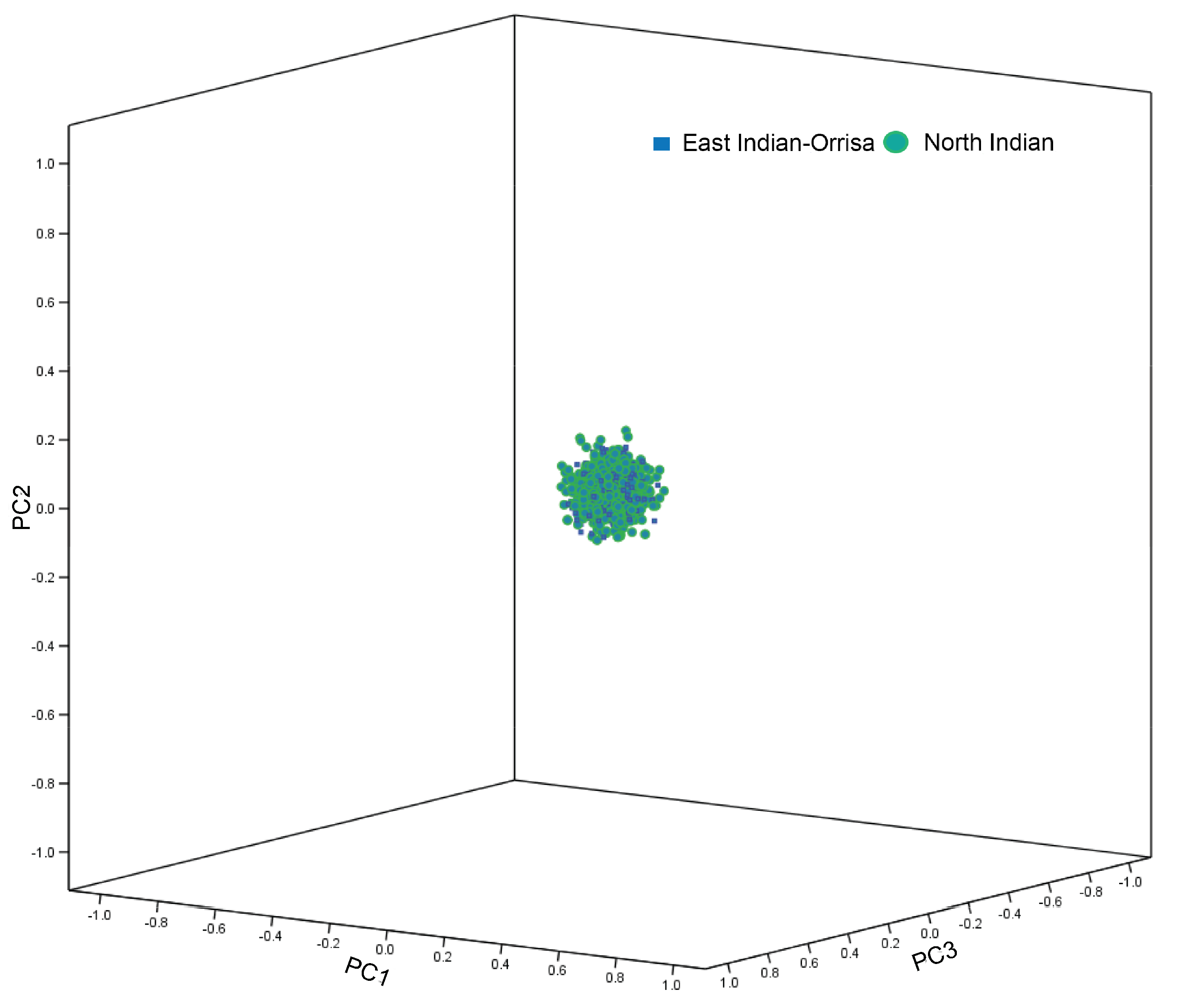

Supplement: Figure S1 — Three dimensional scatter plot showing homogeneity among North Indian and East Indian-Orissa samples. This plot is based on three components generated by principal component analysis. (TIF) [file pgen.1003578.s001.tif]
