# Supplementary material for: Mapping of PARK2 and PACRG Overlapping Regulatory Region Reveals LD Structure and Functional Variants in Association with Leprosy in Unrelated Indian Population Groups
Source: PLoS Genet. 2013 Jul 4;9(7):e1003578. doi: 10.1371/journal.pgen.1003578 (PMC3701713; doi:10.1371/journal.pgen.1003578)
Supplement: Table S1 — Details of 96 SNPs studied in PARK2 and PACRG gene regulatory region in Indian samples (North & East Indian-Orissa population). (DOC) [file pgen.1003578.s002.doc]

**Table S1.** Details of 96 SNPs studied in PARK2 and PACRG gene regulatory region in Indian samples (North & East Indian-Orissa population).

| **S.No.** | **SNP ID** | **Major/Minor Allele** | **Chr Position (Build 37.2)** | **Gene: Region** | **P^a^ Value** | **PAT HWE** | **PAT Minor Allele Frequency** | **CONT HWE** | **CONT Minor Allele Frequency** | **SNP Group1** | **SNP Group2** | **SNP Group3** | **SNP Group4** | **BIN Group1** | **BIN Group2** |
| --- | --- | --- | --- | --- | --- | --- | --- | --- | --- | --- | --- | --- | --- | --- | --- |
| **1** | rs1801334 | G/A | 6:161781225 | PARK2:Exon | NS | 0.88 | 0.005 | 0.84 | 0.01 |  |  |  | Yes | Single SNP BIN | Single SNP BIN |
| **2** | rs56154308 | C/T | 6:162206827 | PARK2:Exon | NW |  |  |  |  |  |  |  | Yes |  |  |
| **3** | rs9456735 | T/G | 6:162475167 | PARK2:Exon | Homo |  |  |  |  |  |  |  | Yes |  |  |
| **4** | rs1801474 | G/A | 6:162622197 | PARK2:Exon | NS | 0.433 | 0.09 | 0.014 | 0.08 |  |  |  | Yes | Single SNP BIN | Single SNP BIN |
| **5** | rs55774500 | C/A | 6:162683724 | PARK2:Exon | Homo |  |  |  |  |  |  |  | Yes |  |  |
| **6** | rs55777503 | AG | 6:162864412 | PARK2:Exon | Homo |  |  |  |  |  |  |  | Yes |  |  |
| **7** | rs1893540 | T/C | 6:163072782 | PARK2:Intron | NS | 0.95 | 0.03 | 0.387 | 0.03 | Yes |  |  |  | BIN-3 | BIN-3 |
| **8** | rs2849515 | G/C | 6:163135233 | PARK2:Intron | NS | 0.44 | 0.16 | 0.167 | 0.16 | Yes |  |  |  | BIN-5 | BIN-5 |
| **9** | rs2849513 | C/G | 6:163135797 | PARK2:Intron | NS | 0.77 | 0.17 | 0.344 | 0.17 | Yes |  |  |  | BIN-5 | BIN-5 |
| **10** | rs2803104 | A/C | 6:163139180 | PARK2:Intron | NS | 0.926 | 0.17 | 0.927 | 0.17 | Yes |  |  |  | BIN-5 | BIN-5 |
| **11** | rs10945859 | T/C | 6:163142602 | PARK2:Intron | 0.00012 | 0.732 | 0.31 | 0.678 | 0.26 | Yes | Yes |  |  | BIN-1 | BIN-1 |
| **12** | rs9347682 | G/A | 6:163144683 | PARK2:Intron | 0.022 | 0.302 | 0.5 | 0.56 | 0.46 |  |  |  | Yes | Single SNP BIN | Single SNP BIN |
| **13** | rs60176253 | T/C | 6:163146905 | PARK2:Intron | NS | 0.871 | 0.04 | 0.274 | 0.03 |  |  |  | Yes | Single SNP BIN | Single SNP BIN |
| **14** | rs62429135 | A/G | 6:163146936 | PARK2:Intron | NS | 0.582 | 0.09 | 0.151 | 0.1 |  |  |  | Yes | Single SNP BIN | Single SNP BIN |
| **15** | rs7746602 | A/A | 6:163147638 | PARK2:Intron | Homo |  |  |  |  |  |  |  | Yes |  |  |
| **16** | rs61173844 | A/C | 6:163148166 | PARK2:Intron;PACRG:5'UTR | NS | 0.487 | 0.18 | 0.231 | 0.2 |  |  |  | Yes | BIN-8 | BIN-8 |
| **17** | rs9347683 (-258) | A/C | 6:163149055 | PARK2:Promoter;PACRG:5'UTR | 0.00018 | 0.512 | 0.31 | 0.438 | 0.26 | Yes |  |  |  | BIN-1 | BIN-1 |
| **18** | rs11538742 | A/C | 6:163149055-163149056 | PARK2:Promoter;PACRG:5'UTR | NW |  |  |  |  |  |  |  | Yes |  |  |
| **19** | rs9456807 | C/C | 6:163149189 | PARK2:Promoter;PACRG:5'UTR | Homo |  |  |  |  |  |  |  | Yes |  |  |
| **20** | rs60168628 | A/A | 6:163149242 | PARK2:Promoter;PACRG:5'UTR | Homo |  |  |  |  |  |  |  | Yes |  |  |
| **21** | rs2276201 | A/G | 6:163149497 | PACRG:Intron | NS | 0.972 | 0.28 | 0.669 | 0.3 | Yes | Yes |  |  | BIN-7 | BIN-7 |
| **22** | rs34044012 | A/C | 6:163150310 | PACRG:Intron | NS | 0.08 | 0.24 | 0.077 | 0.26 |  |  |  | Yes | Single SNP BIN | Single SNP BIN |
| **23** | rs9356058 | T/C | 6:163151399 | PACRG:Intron | NS | 0.577 | 0.17 | 0.158 | 0.16 | Yes | Yes |  |  | BIN-5 | BIN-5 |
| **24** | rs9347684 | T/C | 6:163151824 | PACRG:Intron | 0.00081 | 0.434 | 0.3 | 0.085 | 0.25 | Yes |  |  |  | BIN-1 | BIN-1 |
| **25** | rs6939278 | C/A | 6:163152600 | PACRG:Intron | NS | 0.586 | 0.17 | 0.116 | 0.16 | Yes |  |  |  | BIN-5 | BIN-5 |
| **26** | rs6455848 | G/T | 6:163153238 | PACRG:Intron | NS | 0.794 | 0.18 | 0.238 | 0.2 | Yes | Yes |  |  | BIN-8 | BIN-8 |
| **27** | rs9346929 | G/A | 6:163153373 | PACRG:Intron | 0.00033 | 0.686 | 0.31 | 0.22 | 0.27 | Yes |  |  |  | BIN-1 | BIN-1 |
| **28** | rs10455917 | C/T | 6:163153967 | PACRG:Intron | NS | 0.867 | 0.01 | 0.9 | 0 |  |  |  | Yes | Single SNP BIN | Single SNP BIN |
| **29** | rs13215220 | G/T | 6:163155737 | PACRG:Intron | NW |  |  |  |  |  |  |  | Yes |  |  |
| **30** | rs13195186 | G/A | 6:163159187 | PACRG:Intron | NS | 0.975 | 0.48 | 0.046 | 0.44 |  |  |  | Yes | Single SNP BIN | Single SNP BIN |
| **31** | rs13209100 | G/A | 6:163159350 | PACRG:Intron | NS | 0.242 | 0.1 | 0.202 | 0.1 |  |  |  | Yes | BIN-10 | BIN-10 |
| **32** | rs7764309 | T/G | 6:163162350 | PACRG:Intron | 0.015 | 0.825 | 0.34 | 0.265 | 0.38 |  |  |  | Yes | BIN-11 | BIN-11 |
| **33** | rs13206253 | A/G | 6:163166902 | PACRG:Intron | NS | 0.338 | 0.1 | 0.259 | 0.1 |  |  |  | Yes | BIN-10 | BIN-10 |
| **34** | rs6926597 | T/A | 6:163167157 | PACRG:Intron | NS | 0.106 | 0.02 | 0.647 | 0.01 |  |  |  | Yes | Single SNP BIN | Single SNP BIN |
| **35** | rs9347686 | C/T | 6:163171970 | PACRG:Intron | NS | 0.163 | 0.35 | 0.787 | 0.38 | Yes | Yes |  |  | BIN-4 | BIN-4 |
| **36** | rs4481408 | T/A | 6:163172607 | PACRG:Intron | NS | 0.976 | 0.03 | 0.408 | 0.03 | Yes |  |  |  | BIN-3 | BIN-3 |
| **37** | rs4388273 | G/C | 6:163172809 | PACRG:Intron | NS | 0.976 | 0.03 | 0.367 | 0.03 | Yes |  |  |  | BIN-3 | BIN-3 |
| **38** | rs6926602 | A/A | 6:163173261 | PACRG:Intron | Homo |  |  |  |  |  |  |  | Yes |  |  |
| **39** | rs4709648 | G/C | 6:163173485 | PACRG:Intron | 0.00067 | 0.232 | 0.4 | 0.975 | 0.35 | Yes |  |  |  | BIN-2 | BIN-2 |
| **40** | rs10945861 | T/C | 6:163173678 | PACRG:Intron | NS | 0.169 | 0.36 | 0.612 | 0.38 | Yes | Yes |  |  | BIN-4 | BIN-4 |
| **41** | rs7744306 | T/C | 6:163176151 | PACRG:Intron | NS | 0 | 0.45 | 0 | 0.39 |  |  |  | Yes | Single SNP BIN | Single SNP BIN |
| **42** | rs13210000 | A/G | 6:163176944 | PACRG:Intron | NW |  |  |  |  |  |  |  | Yes |  |  |
| **43** | rs13211655 | C/G | 6:163177542 | PACRG:Intron | 0.018 | 0.606 | 0.33 | 0.799 | 0.37 |  |  |  | Yes | BIN-11 | BIN-11 |
| **44** | rs10755590 | G/A | 6:163180886 | PACRG:Intron | NS | 0.334 | 0.16 | 0.133 | 0.16 |  |  |  | Yes | BIN-5 | BIN-5 |
| **45** | rs9458643 | T/C | 6:163181149 | PACRG:Intron | NS | 0.198 | 0.36 | 0.518 | 0.38 | Yes | Yes |  |  | BIN-4 | BIN-4 |
| **46** | rs9456810 | T/C | 6:163183563 | PACRG:Intron | NS | 0.746 | 0.16 | 0.24 | 0.16 | Yes | Yes |  |  | BIN-5 | BIN-5 |
| **47** | rs12215676 | C/G | 6:163184520 | PACRG:Intron | 0.00012 | 0.209 | 0.4 | 0.512 | 0.34 |  |  |  | Yes | BIN-2 | BIN-2 |
| **48** | rs7742097 | T/C | 6:163187962 | PACRG:Intron | NS | 0.546 | 0.17 | 0.558 | 0.18 | Yes | Yes |  |  | BIN-8 | BIN-8 |
| **49** | rs10806765 | C/T | 6:163190044 | PACRG:Intron | 0.00031 | 0.708 | 0.31 | 0.177 | 0.27 |  |  |  | Yes | BIN-1 | BIN-1 |
| **50** | rs7758074 | A/T | 6:163190838 | PACRG:Intron | NW |  |  |  |  |  |  |  | Yes |  |  |
| **51** | rs10806766 | T/C | 6:163192223 | PACRG:Intron | NS | 0.036 | 0.17 | 0.003 | 0.16 |  |  |  | Yes | BIN-5 | BIN-5 |
| **52** | rs4709650 | A/G | 6:163194173 | PACRG:Intron | NS | 0.398 | 0.04 | 0.467 | 0.03 |  |  |  | Yes | BIN-3 | BIN-3 |
| **53** | rs6936373 | C/G | 6:163195954 | PACRG:Intron | 0.00022 | 0.319 | 0.4 | 0.956 | 0.35 |  |  |  | Yes | BIN-2 | BIN-2 |
| **54** | rs1931223 | T/C | 6:163199086 | PACRG:Intron | NS | 0.825 | 0.17 | 0.298 | 0.17 | Yes | Yes |  |  | BIN-5 | BIN-5 |
| **55** | rs13207363 | G/C | 6:163201285 | PACRG:Intron | 0.03 | 0.65 | 0.33 | 0.231 | 0.36 |  |  |  | Yes | BIN-11 | BIN-11 |
| **56** | rs7759501 | G/T | 6:163201379 | PACRG:Intron | NS | 0 | 0.05 | 0 | 0.04 |  |  |  | Yes | Single SNP BIN | Single SNP BIN |
| **57** | rs13197051 | A/T | 6:163201728 | PACRG:Intron | 0.039 | 0.526 | 0.33 | 0.323 | 0.36 |  |  |  | Yes | BIN-11 | BIN-11 |
| **58** | rs976115 | G/C | 6:163202081 | PACRG:Intron | NS | 0.613 | 0.17 | 0.207 | 0.16 |  |  |  | Yes | BIN-5 | BIN-5 |
| **59** | rs9356060 | A/G | 6:163202778 | PACRG:Intron | NS | 0.517 | 0.18 | 0.217 | 0.17 |  |  |  | Yes | BIN-5 | BIN-5 |
| **60** | rs13212746 | T/C | 6:163203092 | PACRG:Intron | NS | 0.961 | 0.34 | 0.252 | 0.36 |  |  |  | Yes | BIN-11 | BIN-11 |
| **61** | rs11966414 | C/T | 6:163203212 | PACRG:Intron | NS | 0.861 | 0.17 | 0.253 | 0.19 | Yes | Yes |  |  | BIN-8 | BIN-8 |
| **62** | rs13213421 | A/G | 6:163203805 | PACRG:Intron | NS | 0.305 | 0.09 | 0.821 | 0.09 |  |  |  | Yes | BIN-10 | BIN-10 |
| **63** | rs1333956 | G/T | 6:163205091 | PACRG:Intron | NW |  |  |  |  |  |  |  | Yes |  |  |
| **64** | rs1333957 | C/A | 6:163205160 | PACRG:Intron | 0.00013 | 0.693 | 0.31 | 0.408 | 0.26 |  |  |  | Yes | BIN-1 | BIN-1 |
| **65** | rs9458645 | C/T | 6:163207725 | PACRG:Intron | NS | 0.832 | 0.17 | 0.219 | 0.17 | Yes | Yes |  |  | BIN-5 | BIN-5 |
| **66** | rs6915128 | T/C | 6:163211789 | PACRG:Intron | 0.015 | 0.182 | 0.42 | 0.643 | 0.45 | Yes | Yes | Yes |  | BIN-6 | BIN-6 |
| **67** | rs10806767 | C/T | 6:163212299 | PACRG:Intron | NS | 0.832 | 0.17 | 0.192 | 0.17 | Yes | Yes |  |  | BIN-5 | BIN-5 |
| **68** | rs10806768 | A/G | 6:163212414 | PACRG:Intron | 0.017 | 0.196 | 0.41 | 0.72 | 0.45 | Yes | Yes | Yes |  | BIN-6 | BIN-6 |
| **69** | rs9365492 | T/C | 6:163212465 | PACRG:Intron | 0.000015 | 0.634 | 0.31 | 0.111 | 0.25 | Yes | Yes |  |  | BIN-1 | BIN-1 |
| **70** | rs9355403 | G/A | 6:163212578 | PACRG:Intron | 0.00018 | 0.594 | 0.31 | 0.393 | 0.26 |  |  |  | Yes | BIN-1 | BIN-1 |
| **71** | rs1514343 | C/T | 6:163213083 | PACRG:Intron | NS | 0.857 | 0.17 | 0.064 | 0.19 | Yes | Yes |  |  | BIN-8 | BIN-8 |
| **72** | rs1333955 | G/A | 6:163213454 | PACRG:Intron | 0.029 | 0.214 | 0.42 | 0.701 | 0.45 | Yes | Yes | Yes |  | BIN-6 | BIN-6 |
| **73** | rs1040079 | T/C | 6:163214027 | PACRG:Intron | NS | 0.674 | 0.26 | 0.783 | 0.28 | Yes | Yes |  |  | BIN-7 | BIN-7 |
| **74** | rs4495257 | C/A | 6:163214110 | PACRG:Intron | NS | 0.653 | 0.17 | 0.038 | 0.19 | Yes | Yes |  |  | BIN-8 | BIN-8 |
| **75** | rs1514341 | A/G | 6:163216670 | PACRG:Intron | NS | 0.588 | 0.16 | 0.505 | 0.17 |  |  |  | Yes | BIN-12 | BIN-12 |
| **76** | rs9356063 | A/G | 6:163217212 | PACRG:Intron | NS | 0.002 | 0.03 | 0.292 | 0.02 |  |  |  | Yes | Single SNP BIN | Single SNP BIN |
| **77** | rs1041632 | C/T | 6:163219891 | PACRG:Intron | NS | 0.313 | 0.44 | 0.215 | 0.41 | Yes | Yes |  |  | BIN-9 | BIN-9 |
| **78** | rs1041633 | C/T | 6:163220003 | PACRG:Intron | NS | 0.041 | 0.16 | 0.515 | 0.17 |  |  |  | Yes | BIN-12 | BIN-12 |
| **79** | rs1041634 | G/T | 6:163220087 | PACRG:Intron | 0.039 | 0.552 | 0.43 | 0.076 | 0.4 | Yes |  |  |  | BIN-9 | BIN-9 |
| **80** | rs9458647 | G/A | 6:163220429 | PACRG:Intron | 0.035 | 0.265 | 0.44 | 0.483 | 0.41 | Yes | Yes |  |  | BIN-9 | BIN-9 |
| **81** | rs9456812 | A/G | 6:163220490 | PACRG:Intron | NS | 0.426 | 0.43 | 0.026 | 0.39 | Yes | Yes |  |  | BIN-9 | BIN-9 |
| **82** | rs1514335 | A/G | 6:163221548 | PACRG:Intron | NS | 0.274 | 0.41 | 0.003 | 0.37 | Yes |  |  |  | BIN-9 | BIN-9 |
| **83** | rs4458655 | T/C | 6:163221792 | PACRG:Intron | NS | 0.297 | 0.16 | 0.308 | 0.17 |  |  |  | Yes | BIN-12 | BIN-12 |
| **84** | rs6919088 | C/G | 6:163228414 | PACRG:Intron | NS | 0.882 | 0.42 | 0.016 | 0.46 |  |  |  | Yes | BIN-13 | BIN-13 |
| **85** | rs7761529 | C/T | 6:163229582 | PACRG:Intron | NS | 0.054 | 0.04 | 0.815 | 0.04 |  |  |  | Yes | Single SNP BIN | Single SNP BIN |
| **86** | rs6929100 | G/A | 6:163229921 | PACRG:Intron | NS | 0 | 0.06 | 0 | 0.04 |  |  |  | Yes | Single SNP BIN | Single SNP BIN |
| **87** | rs1022275 | A/T | 6:163230141 | PACRG:Intron | NS | 0.092 | 0.34 | 0.021 | 0.31 |  |  |  | Yes | BIN-14 | BIN-14 |
| **88** | rs9346933 | T/C | 6:163231154 | PACRG:Intron | NS | 0.123 | 0.34 | 0.03 | 0.3 |  |  |  | Yes | BIN-14 | BIN-14 |
| **89** | rs1514338 | T/C | 6:163232332 | PACRG:Intron | NS | 0.908 | 0.45 | 0.001 | 0.5 |  |  |  | Yes | BIN-13 | BIN-13 |
| **90** | rs1333960 | G/C | 6:163232983 | PACRG:Intron | NS | 0.916 | 0.42 | 0.014 | 0.46 |  |  |  | Yes | BIN-13 | BIN-13 |
| **91** | rs1333958 | T/G | 6:163233261 | PACRG:Intron | NS | 0.937 | 0.51 | 0.001 | 0.46 |  |  |  | Yes | Single SNP BIN | Single SNP BIN |
| **92** | rs4708974 | A/G | 6:163234467 | PACRG:Intron | NW |  |  |  |  |  |  |  | Yes |  |  |
| **93** | rs4709652 | G/C | 6:163234857 | PACRG:Intron | NS | 0.734 | 0.03 | 0.129 | 0.02 |  |  |  | Yes | Single SNP BIN | Single SNP BIN |
| **94** | rs13199893 | T/C | 6:163235019 | PACRG:Intron | NS | 0.682 | 0.45 | 0.003 | 0.5 |  |  |  | Yes | BIN-13 | BIN-13 |
| **95** | rs34572748 | C/C | 6:163735890 | PACRG:Exon | Homo |  |  |  |  |  |  |  | Yes |  |  |
| **96** | rs60454188 | C/C | 6:163736016 | PACRG:Exon | Homo |  |  |  |  |  |  |  | Yes |  |  |

**NS**-Non-Significant SNPs; **Homo**-Homozygous SNP; **NW**-Not worked SNP

**P^a^ Value** for 2 X 2 chi test for overall allelic frequencies comparison of samples from combined North Indian and East Indian-Orissa population

**SNP Group1**- Common SNPs between Vietnamese (Mira et al and Alter et al) and our study (North, East India-Orissa)

**SNP Group2**- Common SNPs between India-Agra (Alter et al) and our study (North, East India-Orissa)

**SNP Group3**- Common significant SNPs of PARK2 regulatory region between Vietnamese and India-Agra (Alter et al) and our study (North, East India-Orissa)

**SNP Group4**- SNPs exclusive to our Indian study (North, East India-Orissa)

**BIN Group1**- BIN (r^2^>0.08) Details of 96 SNPs studied in our North Indian population

**BIN Group2**- BIN (r^2^>0.08) Details of 96 SNPs studied in our East Indian-Orissa population
