# Supplementary material for: Mapping of PARK2 and PACRG Overlapping Regulatory Region Reveals LD Structure and Functional Variants in Association with Leprosy in Unrelated Indian Population Groups
Source: PLoS Genet. 2013 Jul 4;9(7):e1003578. doi: 10.1371/journal.pgen.1003578 (PMC3701713; doi:10.1371/journal.pgen.1003578)
Supplement: Table S2 — Allele frequencies for 11 significant SNPs within PARK2 and PACRG gene regulatory region along with their respective P Values and Odds Ratio in East Indian-Orissa population. (DOC) [file pgen.1003578.s003.doc]

**Table S2.** Allele frequencies for 11 significant SNPs within PARK2 and PACRG gene regulatory region along with their respective *P* Values and Odds Ratio in East Indian-Orissa population.

| **SNP** | **Risk Allele** | **Patients** | **Control** | **p^a^ Value** | **OR (95%CI)** |
| --- | --- | --- | --- | --- | --- |
| rs10945859 | Minor-C | 0.23 | 0.16 | 0.013 | 1.58(1.1-2.27) |
| rs9347683 | Minor-C | 0.23 | 0.16 | 0.017 | 1.56(1.08-2.24) |
| rs9347684 | Minor-C | 0.22 | 0.14 | 0.0057 | 1.7(1.16-2.48) |
| rs9346929 | Minor-A | 0.23 | 0.15 | 0.0047 | 1.7(1.18-2.47) |
| rs4709648 | Minor-C | 0.31 | 0.22 | 0.0064 | 1.57(1.14-2.18) |
| rs12215676 | Minor-G | 0.31 | 0.22 | 0.0079 | 1.56(1.12-2.16) |
| rs10806765 | Minor-T | 0.23 | 0.15 | 0.0039 | 1.72(1.19-2.49) |
| rs6936373 | Minor-G | 0.31 | 0.22 | 0.0089 | 1.55(1.12-2.15) |
| rs1333957 | Minor-A | 0.23 | 0.15 | 0.0031 | 1.75(1.21-2.55) |
| rs9365492 | Minor-C | 0.23 | 0.15 | 0.0038 | 1.73(1.19-2.52) |
| rs9355403 | Minor-A | 0.23 | 0.15 | 0.0026 | 1.77(1.22-2.56) |

**p^a^** value for 2 X 2 chi test for overall allelic frequencies comparison between case controls in East Indian-Orissa population, Odd’s ratio and 95% confidence interval.
