# Supplementary material for: Mapping of PARK2 and PACRG Overlapping Regulatory Region Reveals LD Structure and Functional Variants in Association with Leprosy in Unrelated Indian Population Groups
Source: PLoS Genet. 2013 Jul 4;9(7):e1003578. doi: 10.1371/journal.pgen.1003578 (PMC3701713; doi:10.1371/journal.pgen.1003578)
Supplement: Table S3 — Allele combinations generated in four Clones for 2 SNPs, located upstream of the PARK2 gene regulatory region, and their Bioinformatics prediction for transcription factor binding by using TRANSFEC and HaploReg databases. (DOC) [file pgen.1003578.s004.doc]

**Table S3.** Allele combinations generated in four Clones for 2 SNPs, located upstream of the PARK2 gene regulatory region, and their Bioinformatics prediction for transcription factor binding by using TRANSFEC and HaploReg databases.

|  |  | **Transcription factors binding prediction by Tansfac-AliBaba2 tool** | | **Result of HeploReg database** | |
| --- | --- | --- | --- | --- | --- |
| **CLONE** | **Allele combination** | **rs9365492 (T/*C)** | **rs9355403 (G/*A)** | **rs9365492 (T/*C)** | **rs9355403 (G/*A)** |
| Clone1 | rs9365492(T)-rs9355403(G) |  | C/EBPalp,HNF-1,GATA-1,C-EBPbeta | Sox,XBP-1 | Foxa |
| Clone2 | rs9365492(*C)-rs9355403(G) | ATF,CRE-BP1 | C/EBPalp,HNF-1,GATA-1,C-EBPbeta |  | Foxa |
| Clone3 | rs9365492(T)-rs9355403(*A) |  |  | Sox,XBP-1 |  |
| Clone4 | rs9365492(*C)-rs9355403(*A) | ATF,CRE-BP1 |  |  |  |

*****Alleles represent the risk allele for the SNP.
