# Supplementary material for: Mapping of PARK2 and PACRG Overlapping Regulatory Region Reveals LD Structure and Functional Variants in Association with Leprosy in Unrelated Indian Population Groups
Source: PLoS Genet. 2013 Jul 4;9(7):e1003578. doi: 10.1371/journal.pgen.1003578 (PMC3701713; doi:10.1371/journal.pgen.1003578)
Supplement: Table S4 — Allele frequency comparison of 2 Significant SNPs (rs9365492 and rs9355403) between different HapMap and our North-Indian populations. (DOC) [file pgen.1003578.s005.doc]

**Table S4.** Allele frequency comparison of 2 Significant SNPs (rs9365492 and rs9355403) between different HapMap and our North-Indian populations.

| **Population** | **Details** | **rs9365492 (Major-T)** | **rs9365492 (Minor- Risk-C)** | **rs9355403 (Major-G)** | **rs9355403 (Minor- Risk-A)** |
| --- | --- | --- | --- | --- | --- |
| **YRI** | Sub-Saharan African | 0.991 | 0.009 | 0.95 | 0.05 |
| **CEU** | European | 0.816 | 0.184 | 0.805 | 0.195 |
| **OUR** | North Indian | 0.731 | 0.269 | 0.724 | 0.276 |
| **CHB** | Asian | 0.443 | 0.557 | 0.456 | 0.544 |
| **JPT** | Asian | 0.474 | 0.526 | 0.5 | 0.5 |
