# Supplementary material for: Mapping of PARK2 and PACRG Overlapping Regulatory Region Reveals LD Structure and Functional Variants in Association with Leprosy in Unrelated Indian Population Groups
Source: PLoS Genet. 2013 Jul 4;9(7):e1003578. doi: 10.1371/journal.pgen.1003578 (PMC3701713; doi:10.1371/journal.pgen.1003578)
Supplement: Table S5 — Primer sequences along with restriction sites for cloning specific regions of PARK2 and PACRG. (DOC) [file pgen.1003578.s006.doc]

**Table S5.** Primer sequences along with restriction sites for cloning specific regions of PARK2 and PACRG.

| **Upstream of PACRG gene containing SNP rs10945859** | | **Restriction site** |
| --- | --- | --- |
| Forward | 5'-AAAAGCTAGCTTATCTGGACTTGCTGGCACT-3' | Nhe1 |
| Reverse | 5'-AAAACTCGAGCGTTGATCTGAATGCCCTTT-3' | Xho1 |
| **Upstream of PARK2 gene containing SNP rs9347684** | |  |
| Forward | 5'-AAAAGCTAGCTGTTTCATCAAATGGGCTGT-3' | Nhe1 |
| Reverse | 5'-AAAACTCGAGTTGGACCACTTCAGGTTTCA-3' | Xho1 |
| **Upstream of PARK2 gene containing rs9365492 and rs9355403** | |  |
| Forward | 5'-AAAAGCTAGCGAGAGAAGCAGCCTCCCTATT-3' | Nhe1 |
| Reverse | 5'-AAAACTCGAGCCGTCAGCATGTGAAACATC-3' | Xho1 |
